# Prenatal alcohol exposure increases the aggressiveness of estrogen‐induced pituitary tumors in male rats

**DOI:** 10.1111/acer.70169

**Published:** 2025-09-29

**Authors:** Shaista Chaudhary, Dipak K. Sarkar

**Affiliations:** ^1^ Endocrinology Program Rutgers, The State University of New Jersey New Brunswick New Jersey USA; ^2^ Department of Animal Sciences Rutgers, The State University of New Jersey New Brunswick New Jersey USA

**Keywords:** aggressive pituitary tumors, cell stemness, developmental pluripotency associated 4 gene, Fischer male rats, prenatal alcohol exposure

## Abstract

**Background:**

We have recently shown that estrogen‐induced prolactin‐secreting pituitary tumors are aggressive in prenatal alcohol‐exposed female rats. In this study, we investigated whether similar tumor aggressiveness occurs in estrogen‐treated prenatal alcohol‐exposed male rats.

**Methods:**

Pregnant Fischer 344 rats were fed from gestational days 7 and 21 with a liquid diet containing ethanol 6.7% v/v (AF), pair‐fed with an isocaloric liquid diet (PF), or fed chow ad libitum (AD). Alcohol‐fed dams exhibited a blood alcohol concentration of 120–150 mg/dL 2 h after the last feeding. Male offspring were orchiectomized at 60 days of age and implanted subcutaneously with estradiol implants. Four months after the estradiol implants, rats were sacrificed, and pituitary tumor tissues were collected. Tumor cells were isolated and cultured for analysis.

**Results:**

Pituitary tumor cells from AF males exhibited stem‐like cell properties and showed elevated expression of stem cell regulatory genes and proteins (SOX‐2, OCT‐4, KLF4, SNAIL‐1, and Nestin), tumor aggressiveness markers (MMP‐9, CD44, CD34, PTTG, FGFR4, Ki‐67, N‐Cadherin), and prolactin compared to those from AD and PF controls. AF cells also had a higher cell proliferation rate, increased invasiveness, and colony formation compared to those in AD and PF cells, indicating more aggressive cancer cells than control cells. Notably, AF cells had a higher expression of developmental pluripotency‐associated 4 (Dppa4), a gene we recently identified as upregulated in aggressive tumors and in fetal alcohol‐exposed animals.

**Conclusions:**

These findings are consistent with our previous observations in estrogen‐treated AF female rats. These results support the hypothesis that prenatal alcohol exposure programs the pituitary epithelium toward a mesenchymal stem cell‐like phenotype, contributing to the development of aggressive pituitary prolactinomas in both sexes.

## Graphical abstract

Prenatal alcohol exposure (PAE) enhances the development of aggressive pituitary tumors (PitNETs) in female rats. In this study, we found that PAE also promotes the development of PitNETs in male rats. Like in females, PAE treatment enhances the expression of stem cell and tumor aggressiveness genes and increases tumor cell proliferation rate, invasiveness, and colony formation in the pituitary. These findings suggest that PAE reprograms pituitary cells toward a mesenchymal stem cell‐like state, promoting aggressive PitNET development in both sexes.

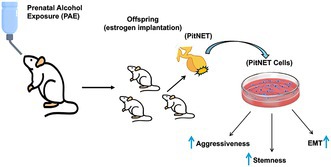

## INTRODUCTION

Pituitary adenomas and pituitary carcinomas are now referred to as pituitary neuroendocrine tumors (PitNET). These tumors are the most common intracranial neoplasm, accounting for approximately 10% of the total. Over 65% of these tumors are secretory, and half of them are prolactin‐secreting (prolactinoma), which are known to cause amenorrhea‐galactorrhea and hypogonadism in humans (Trouillas et al., 2020). Some of these tumors demonstrate a lack of sensitivity to dopamine therapy and often exhibit gross invasion of the surrounding tissues or distant metastasis (Neou et al., 2020; Raverot et al., 2018). The etiology of aggressive PitNETs is not apparent and requires further investigation (Das et al., 2021). Stem cell involvement has recently been suggested because the activated phenotype of pituitary‐resident stem cells is present in human tumors (Mertens et al., 2015; Nys et al., 2022), and pituitary stem cells have been shown to be resistant to dopamine agonist treatment (Cai et al., 2021).

We have used an animal model in which prenatal alcohol exposure (PAE) increased the expression of prolactin in the pituitary and induced aggressive PitNET following exogenous estradiol treatment by increasing pituitary cell stemness in female rat offspring (Chaudhary et al., 2025; Jabbar et al., 2018). In this animal model, tumor cells formed spheres when cultured (tumorispheres) in an ultra‐low attachment plate and expressed stem cell marker genes. These tumorispheres formed solid tumors in immunodeficient mice (Jabbar et al., 2018), identifying their stem cell characteristics (Clevers, 2011). Thus, the estrogen‐treated PAE female rat model is useful to identify important molecular pathways regulating stemness and tumor aggressiveness in the pituitary. However, it is not known whether exogenous estradiol similarly increases pituitary cell stemness and induces aggressive PitNETs in the pituitary of male PAE rats. The presence of estrogen in males has been documented, and this steroid has been shown to play an essential role in male physiology (Cooke et al., 2017; Hess & Cooke, 2018). Furthermore, estrogen's influence on pituitary progenitor stem cells in the development of prolactinoma has been described in males (García Barrado et al., 2014, 2016). We show here that PAE increased pituitary cell stemness, stemness‐inducing factor DPPA4 and its signaling molecules, and induced aggressive PitNET following exogenous estradiol treatment in male offspring.

## MATERIALS AND METHODS

### Animal model

To perform this research, we used an animal model of PAE that was adopted from our previously published studies (Chaudhary et al., 2025; Jabbar et al., 2018). We assessed the induction of heritable epigenetic cell stemness variants by fetal alcohol exposure in the pituitary of isogenic F344 rats maintained in standardized conditions to control both genetic and environmental sources of variations (Gangisetty et al., 2022). We purchased female Fischer‐344 rats (5–6 weeks; 80–100 g) from Harlan Laboratories (Indianapolis, IN, USA) and housed them in pairs in open‐type shoebox cages with Bedcob bedding. Animals were maintained in a room under the controlled condition of a 12‐h light/dark cycle (6:00 am/6:00 pm). Healthy adult female rats showing regular estrous cycles were bred with fertile adult male rats. On gestational day 7 through 21, animals were fed either rat chow ad libitum (AD), a liquid diet containing ethanol (AF), or pair‐fed (PF) an isocaloric liquid control diet (with ethanol calories replaced by maltose‐dextrin). The concentration of ethanol in the diet was increased over the first 4 days from 1.7% to 5.0% v/v to habituate the animals to the alcohol diet. After this habituation period, rats were fed the liquid diet containing ethanol at a concentration of 6.7% v/v. On postnatal day 2 (PD2), AF and PF pups were cross‐fostered to untreated lactating AD dams to prevent any compromised nurturing by the AF and PF mothers. Litter size was maintained at eight pups per dam to minimize any nurturing effect on the body growth. Pups were weaned on PD21 and housed by sex. At the age of 60 days (weight 130–150 g), male grown‐up pups were castrated and subcutaneously (sc) implanted with an estradiol‐17β (Sigma‐Aldrich) containing silastic capsule (1 cm; Dow Corning) under 2% isoflurane anesthesia and 2.5% bupivacaine sc to induce local analgesia. Analgesic drug treatment was continued for 3 days after surgery for the prevention of pain. Estradiol‐containing silastic capsules are known to maintain a constant physiological level of the steroid in plasma for a long period (Isaksson et al., 2011) and induce pituitary tumors (Sarkar, 2006). This tumor model is accepted as a valid preclinical animal model for prolactinomas (Šošić‐Jurjević et al., 2020).

After 120 days of estradiol implants, rats were euthanized, and their pituitary tumor tissues were collected and used for experimentation. All surgical procedures and euthanasia procedures were in accordance with Rutgers institutional guidelines and use committee‐approved research protocols.

### Isolation and culture of pituitary tumor cells

Primary cultures of rat pituitary tumor cells were prepared using a published method with minor modifications (Chang et al., 2017; Chaudhary et al., 2025). Briefly, pituitary tumor tissue was washed with PBS to remove all the blood, minced the tissue, and then digested using 10 mg/mL collagenase (Sigma‐Aldrich) and 0.5% DNAase (Sigma‐Aldrich) in HBSS (Gibco) for 20 min at 37°C with frequent pipetting using a glass Pasteur pipette. The homogenous cell suspension was filtered through a 40‐*μ*m cell strainer and centrifuged at 376 × g for 10 min. Cells were pelleted, resuspended, and loaded on top of discontinuous density gradients consisting of 35%, 50%, and 60% Percoll layers, centrifuged at 376 × g for 20 min, and the lactotroph‐enriched Percoll layer between 35% and 50% was collected. Cells were dissociated, counted, and plated into poly‐l‐lysine (Sigma‐Aldrich)‐coated six‐well plates. Cells were maintained for 2 days in high‐glucose Dulbecco's modified Eagle's medium (DMEM) supplemented with 1% Penicillin and Streptomycin, 2.5% fetal bovine serum (FBS; Gibco), 10% horse serum (Gibco), and l‐glutamine (Sigma‐Aldrich), and then maintained in serum‐free DMEM containing insulin (5 *μ*M), putrescine (1 *μ*M), transferrin (100 *μ*M), and sodium selenite (30 nM) at 37°C in a humidified atmosphere of 7.5% CO_2_.

### Determination of plasma prolactin

To determine the concentration of prolactin (PRL) in plasma, blood samples were collected from AD, PF, and AF group rats and centrifuged at 1503 × g for 10 min; plasma was collected and used to measure the levels of PRL by using the rat PRL EIA kit (Alpco Diagnostics, Salem, NH, USA) according to the instructions from the manufacturer. We used 10 *μ*L of plasma sample in duplicates for PRL measurements.

### Cell proliferation assay

Cell proliferation rate was determined using MTT assay according to our previous study (Chaudhary et al., 2025; Jabbar et al., 2018). In brief, rat pituitary tumor cells were seeded in 96‐well plates at a cell density of 3 × 10^3^/well or 4 × 10^3^/well in triplicates and grown for 24, 48, and 72 h. Cell proliferation rate was measured at 0, 24, 48, and 72 h by a colorimetric assay based on the conversion of the water‐soluble yellow dye MTT [3‐(4,5‐dimethylthiazol‐2‐yl)‐2,5‐diphenyltetrazolium bromide] (Sigma‐Aldrich) to an insoluble purple formazan by the action of mitochondrial reductase enzyme. Formazan is then solubilized, and the concentration was determined by absorbance at 595 nm on a Multiskan FC (Thermo Fisher Scientific).

### Trans‐well migration assay

Trans‐well migration assay was performed in a 24‐well plate setup using 8.0‐*μ*m pore size inserts as previously described (Chaudhary et al., 2025). Rat pituitary tumor cells at a density 5 × 10^5^ were plated in the upper compartment of the inserts. Lower compartments contained DMEM with 0.5% FBS, 0.25% horse serum, and 40 *μ*g/mL collagen (Sigma‐Aldrich). After 24 h, inserts were harvested, clearing the remaining cells in the upper compartment using cotton swabs, and fixed in 5% glutaraldehyde for 10 min. Next, the inserts were stained with 0.5% crystal violet (Sigma‐Aldrich) for 15 min; then, inserts were washed with distilled water and left to dry before imaging. Migrated cells were counted using the ImageJ analysis software (National Institutes of Health).

### Colony formation assay

2 × 10^3^ cells/well were seeded into six‐well plates for colony formation assay. Colonies formed in each well after 14 days, were washed with PBS and fixed with cold methanol at −20°C for 10 min and stained with 0.5% crystal violet (Sigma‐Aldrich) for 15 min. The number of colonies formed was counted by using the ImageJ analysis software (National Institutes of Health).

### Immunocytochemistry

Immunocytochemical analysis was performed according to the method described by us previously (Jabbar et al., 2018; Chaudhary et al., 2025). Rat pituitary tumor cells were fixed in 4% paraformaldehyde for 15 min. Fixed cells were rinsed two times with PBS and permeabilized with Triton X‐100 (0.2% or 0.4% for cytoplasmic and nuclear staining, respectively). Fixed cells were incubated in blocking buffer containing 5% normal donkey/goat serum in PBS for 30 min at room temperature, and then, cells were incubated with primary antibodies (diluted in blocking buffer) overnight at 4°C. The next day, cells were incubated with fluorescence‐tagged secondary antibodies diluted in blocking buffer and for 1 h at room temperature in the dark, followed by three washes with PBS. Nuclei were stained with 0.1 *μ*g/mL DAPI (Sigma‐Aldrich). Cells were mounted using Vecta‐Shield antifade mounting media (H‐1200; Vector Laboratories). Fluorescent images were acquired using a Nikon‐TE 2000 inverted microscope (Nikon Instruments).

### RT‐PCR (qPCR)

The c‐DNA was prepared using 1 *μ*g of RNA from each sample, and a 1:3 dilution was done using nuclease‐free water. The real‐time qPCR was carried out in 384‐well plates. Each well of the 384‐well plates contains a reaction mixture of 10 *μ*L consisting of a 1:3 dilution of c‐DNA, primers (for more information, see Chaudhary et al., 2025) in Power SYBR Green PCR Master Mix, and the comparative *C*_T_ (ΔΔ*C*_T_) standard run method was carried out in the Quant Studio 7 real‐time PCR system. The expression of genes was expressed in terms of relative quantification using the formula $2^{-\Delta \Delta C_{T}}$ (Rousseau et al., 2022).

### Immunoblotting

Total protein was extracted from tissues or cells using RIPA lysis buffer supplemented with a protease and phosphatase inhibitor cocktail (Thermo Fisher Scientific). Protein concentration was measured using Bradford reagent protein assay kit (Bio‐Rad). Thirty to fifty microgram protein was electrophoresed through SDS‐PAGE and transferred onto polyvinyldifluoride (PVDF) membranes (Bio‐Rad). Membranes were blocked using 5% nonfat dry milk or 5% BSA as per the optimized conditions and incubated with primary antibodies overnight at 4°C and washed with 1× TBS‐T and incubated with species‐specific horseradish peroxidase‐conjugated secondary antibody for 1 h at room temperature. After that, membranes were subsequently washed and developed using an enhanced chemiluminescence ECL substrate (Thermo Fisher Scientific) on the film or iBright 1500 (Invitrogen). The intensity of individual bands on immunoblots was measured by the ImageJ software and normalized with loading control.

### Subcutaneous xenograft experiments

NOD/SCID mice (Charles River Laboratories) were used for xenograft studies as previously described (Chaudhary et al., 2025; Jabbar et al., 2018). Briefly, 1 × 10^6^ rat pituitary tumor cells were subcutaneously injected with an equal volume of Matrigel (354248; Corning) in the right flank of animals. Tumors were measured using electronic calipers, and tumor volumes were calculated as *V* = *L* × *W* ^2^ × 0.5. The mean ± SEM tumor volume was calculated every alternate day or twice a week for each experimental group and presented. After sacrificing the animals, the tumors were excised, weighed, sized, and photographed to visualize differences in tumor morphology. For the survival experiments, mice were sacrificed when signs of morbidity that met ethical criteria for sacrifice were observed or when the tumor reached an average size of 4000–5000 mm^3^, and the survival curves were plotted in a Kaplan–Meier survival curve.

## RESULTS

### Prenatal alcohol exposure increases tumor growth and expression of stem‐like cells in the pituitary

**FIGURE 1:**
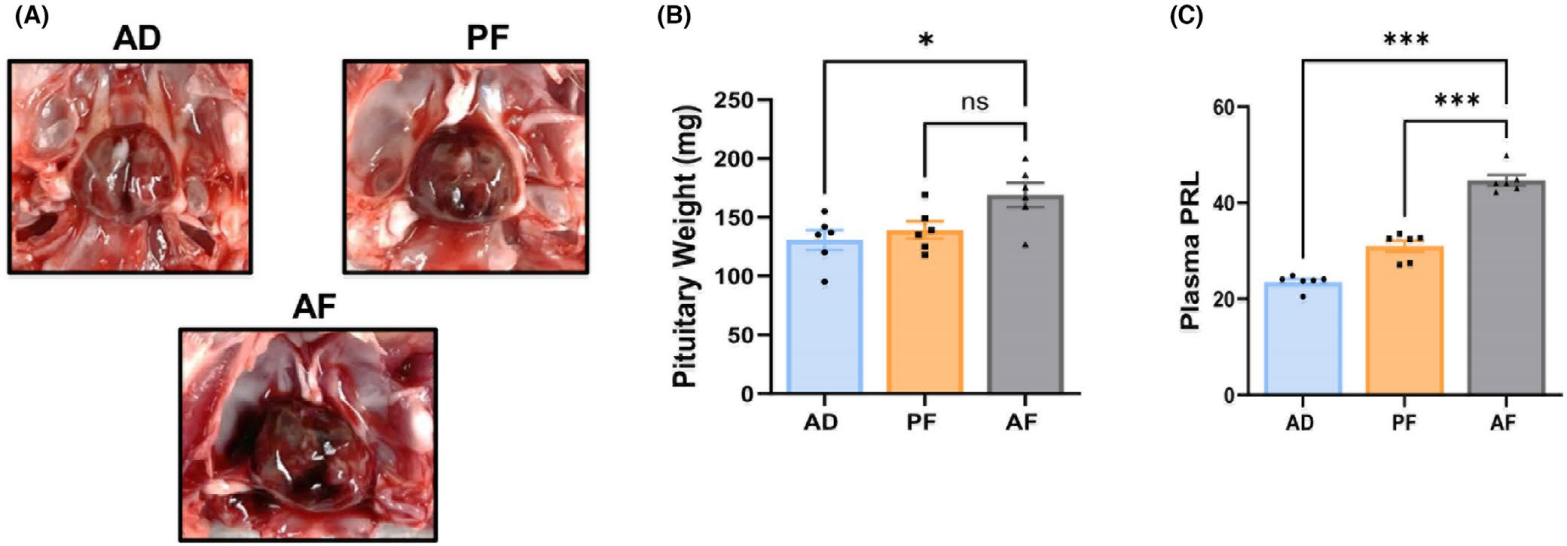
Effects of fetal alcohol exposure on pituitary morphology, weight, and plasma PRL. Representative photographs of the pituitary tumors are shown (A), pituitary weight (B), and plasma PRL (C) in alcohol‐fed (AF), ad libitum‐fed (AD), and pair‐fed (PF) rats. Data are mean ± SEM (*n* = 6) and were analyzed using one‐way analysis of variance (ANOVA) with the Newman–Keuls post hoc test. *p < 0.05, and ***p < 0.001 between AF and controls (AD, PF).

**FIGURE 2:**
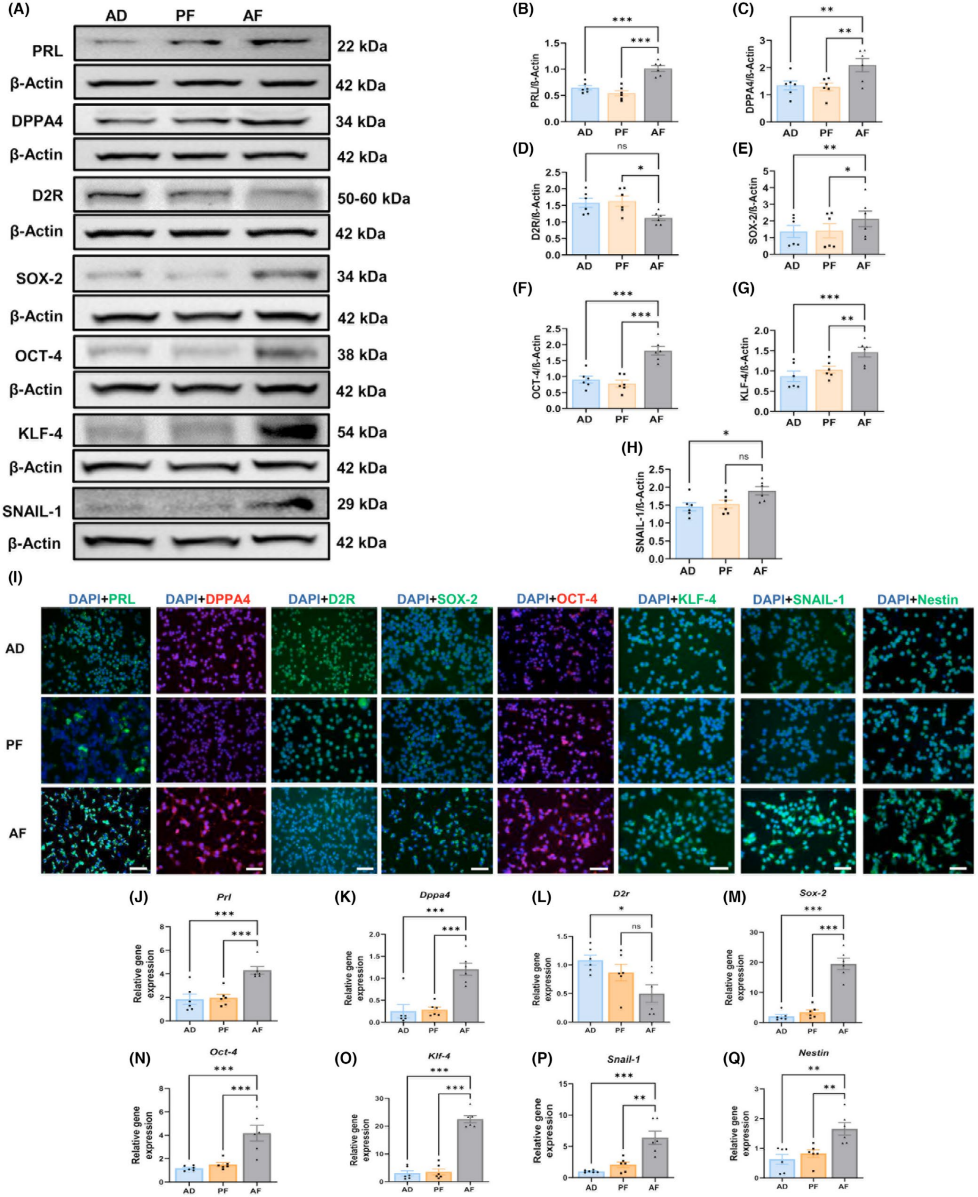
Expression differences of stem cell regulatory proteins in the pituitary between AF, PF, and AD rats treated with estradiol. Protein levels were measured by western blotting (A) and immunocytochemistry (B), and mRNA expression of by qPCR (C). Western botting protein expression of PRL (B), DPPA4 (C), D2R (D), SOX‐2 (E), OCT‐4 (F), KLF‐4 (G), SNAIL‐1 (H), and mRNA expression of PRL (J), DPPA4 (K), D2R (L), SOX‐2 (M), OCT‐4 (N), KLF‐4 (O), SNAIL‐1 (P), Nestin (Q) in pituitary tumor cells of AF, PF and AD rats. Data are mean ± SEM (*n* = 6) and were analyzed using one‐way analysis of variance (ANOVA) with the Newman–Keuls post hoc test. *p < 0.05, **p < 0.01, and ***p < 0.001 between AF and controls. Scale bar represents 100 *μ*M.

Consistent with what we have reported in female PAE rats, we show here that in male rats, estrogen promoted the development of pituitary tumors in AF rats in relation to size (Figure 1A) and weight (Figure 1B) when compared with those in AD and PF rats. AF rats also showed a significant increase in the level of plasma PRL (Figure 1C) when compared with those in AD and PF rats. Also consistent with female rats, cells derived from the tumors of AF‐treated male rats showed higher expression of PRL and DPPA4, lower expression of D2R, and increased expression of stem cell regulatory proteins (SOX‐2, OCT‐4, KLF4, SNAIL‐1, and Nestin) when compared with those in AD and PF tumor cells, as determined by western blot (Figure 2A–K) and immunofluorescence (Figure 2I) for proteins and qPCR for genes (Figure 2J–Q). These results indicate that pituitary tumor cells of AF rats have expanded stem cell niches.

### Fetal alcohol exposure increases expression of tumor aggressiveness markers

**FIGURE 3:**
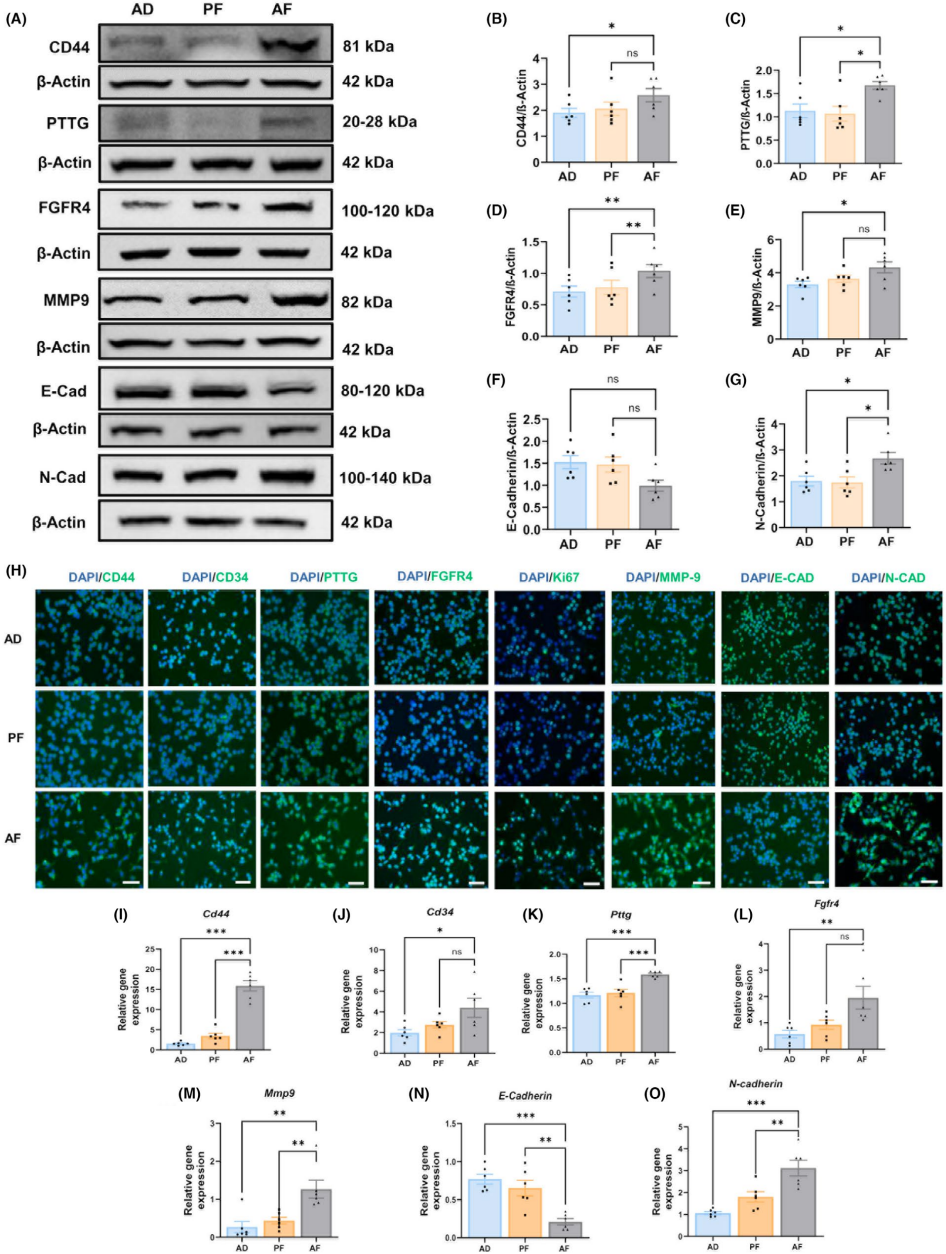
Expression differences of tumor aggressiveness proteins in the pituitary between AF, PF, and AD rats treated with estradiol. Protein levels were measured by western blotting (A) and immunocytochemistry (H), and mRNA expression was measured by qPCR (C). Western botting protein expression of CD44 (B), PTTG (C), FGFR4 (D), MMP9 (E), E‐Cadherin (F), N‐Cadherin (G), mRNA expression of CD44 (I), CD34 (J), PTTG (K), FGFR4 (L), MMP9 (M), E‐Cadherin (N), N‐Cadherin (O) in pituitary tumor cells of AF, PF and AD rats. Data are mean ± SEM (*n* = 6) and were analyzed using one‐way analysis of variance (ANOVA) with the Newman–Keuls post hoc test. *p < 0.05, **p < 0.01, and ***p < 0.001 between AF and controls. Scale bar represents 100 *μ*M.

As shown in previous studies, pituitary tumors are generally adenomas, but some, including PAE female rat pituitary tumors, demonstrate aggressive and/or malignant behavior (Chaudhary et al., 2025; Jabbar et al., 2018). We have also examined the expression of several tumor aggressiveness markers (MMP‐9, CD44, CD34, PTTG, FGFR4, Ki‐67, E‐Cadherin, and N‐Cadherin) in AF, AD, and PF tumor cells of male rat pituitaries (Figure 3). The expression of all tumor aggressiveness markers was elevated in AF pituitary tumor cells and decreased expression of E‐Cadherin when compared with PF and AD pituitary tumor cells, as determined by western blotting (Figure 3A–H) and immunofluorescence (Figure 3I) for proteins and qPCR for genes (Figure 3J–Q).

### Fetal alcohol exposure increases tumorigenicity in pituitary tumor cells

**FIGURE 4:**
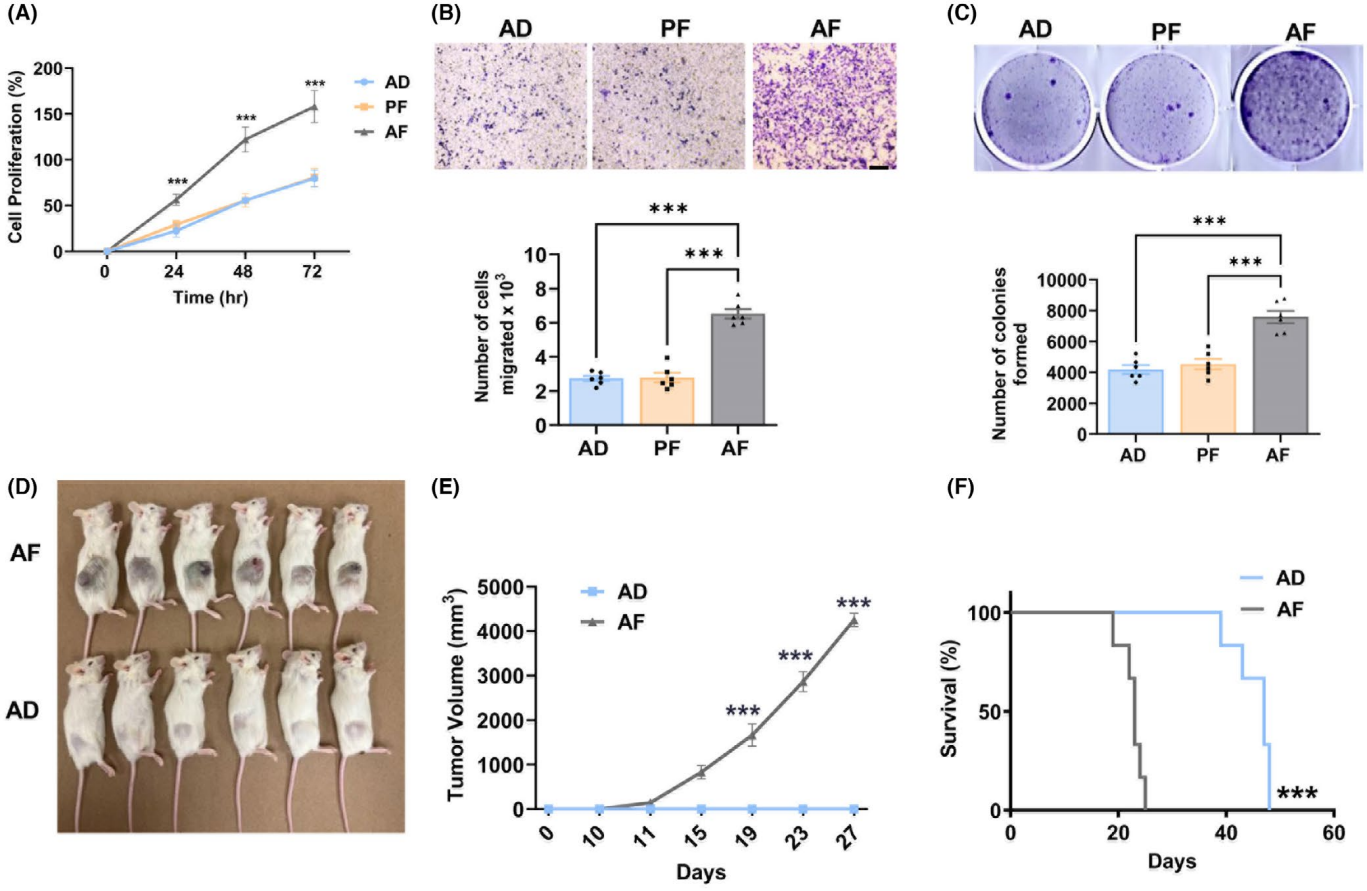
Effects of fetal alcohol exposure on cell proliferation, colony formation, migration, and tumorigenic ability of pituitary tumor cells. Cell proliferation rate (A), cell migration rate (B), and colony formation rate (C). Pictures of animals with tumors in AF and AD groups (D), tumor volume changes (E), and effects on survival time (F). Data are mean ± SEM (*n* = 6) and were analyzed using one‐way analysis of variance (ANOVA) with the Newman–Keuls post hoc test. ***p < 0.001 between AF and controls (AD, PF). Kaplan–Meier survival analysis was used to test significant differences between survival curves and mean survival time for mice from each group.

To determine the growth rate of AF, AD, and PF pituitary tumor cells, we conducted cell proliferation, migration, and colony formation assays. For the cell proliferation assay, AF, AD, and PF cell proliferation rates were measured at 0, 24, 48, and 72 h. As shown in Figure 4A, there is no significant difference in the cells' proliferation rate between AD and PF cells at any of the recorded times. When compared between AF and AD or PF cells, there was a significant difference between the cell proliferation rates of these groups. AF pituitary tumor cells proliferated at least double the rate compared to AD and PF pituitary tumors. AF pituitary tumor cells also had a higher migration and colony formation rate when compared with AD and PF pituitary tumor cells, as shown in Figure 4B,C.

We also examined the tumorigenic potential of the pituitary tumor cells by inoculating the AF and AD cells in NON/SCID immunodeficient mice and observed that AF cells formed large tumors after 3 weeks of cell grafting (Figure 4D) with a rapid growth rate (Figure 4E), whereas AD pituitary tumor cells did not form any tumor growth. The survival rate analysis following cell xenografts showed that the lifespan of mice injected with AF cells significantly decreased when compared with AD cells (Figure 4F).

## DISCUSSION

The data presented here indicates that PAE in male rats increases both pituitary weight and PRL responses to estrogen compared with those in control groups. Inspection of the pituitary gland after estrogen treatment revealed that some tumors in PAE rats were highly vascularized, penetrated the sphenoid bone, and compressed the median eminences. These findings suggest that PAE male rat pituitaries are more responsive to the mitogenic action of estrogen than those of control male rat pituitaries. Similar effects of estrogen were observed previously in AF pituitaries of female rats (Chaudhary et al., 2025; Jabbar et al., 2018).

Comparison between protein and gene transcript levels in the pituitary of PAE male rats and control male rats indicated that PAE rat pituitaries had higher levels of PRL, suggesting that these tumors are primarily prolactinomas. Similar to the previous findings in PAE female rat pituitary (Chaudhary et al., 2025; Jabbar et al., 2018), PAE male rat pituitary also showed decreased D2R levels, an inhibitory regulator of prolactin synthesis and release (Cristina et al., 2006; Sarkar, 2006). Moreover, the ethanol‐responsive oncogenic factor DPPA4, which is overexpressed in various cancers (Klein & Knoepfler, 2021; Li et al., 2019; Tung et al., 2013), was elevated in PAE male rat pituitaries following estrogen treatment (Chaudhary et al., 2025). DPPA4 has been shown to increase stem cell regulatory proteins and promote tumor cell stemness in PAE female rat pituitaries (Chaudhary et al., 2025). In this study, we found higher protein and/or mRNA levels of DPPA4‐associated stem cell factors (SOX‐2, OCT‐4, KLF‐4, SNAIL‐1, and Nestin) in PAE male pituitaries (Chaudhary et al., 2025), suggesting that PAE similarly enhances estrogen‐induced pituitary tumor cell stemness in both sexes.

Upregulation of the SOX2‐positive pituitary stem cells by transgenic approaches stimulates a transient proliferation burst and subsequently induces tumorigenesis in a noncell autonomous manner (Andoniadou, 2016). OCT4 and SOX2 are established markers of embryonic and pituitary stem cells (Chang et al., 2017), and their presence in pituitary adenomas has also been demonstrated (Levy, 2008). Additionally, rat prolactinoma cells also contained OCT4‐ and SOX2‐positive cells (Gao et al., 2017). Expression of multipotency‐related genes (OCT4, KLF4, SOX2, CD133, Nestin, and Snail) was significantly higher in pituispheres from AF female rats than those from AD female rats (Jabbar et al., 2018). Therefore, our results showing increased SOX2, OCT4, and other stem cell markers in PAE male pituitary tumors suggest that overexpression of stem cell markers, which may contribute to their aggressiveness.

The aggressiveness of pituitary tumors in PAE male rats was further evaluated by measuring several biomarkers. We observed that the Ki67 immunostaining level was elevated in PAE pituitary tissue. Ki‐67 provides a nuclear labeling index and is the most reliable marker for distinguishing proliferating from quiescent cells (Onguru et al., 2004). Recent evidence indicates that CD44, CD34, PTTG, FGFR4, MMP9, E‐Cadherin, and N‐Cadherin serve as biomarkers of aggressive pituitary adenomas (Mete et al., 2012; Valea et al., 2022). CD44 has been identified as a molecular marker of invasive pituitary adenomas (Moldovan et al., 2017) and is present in cancer stem‐like cells in endocrine tissues (Lloyd et al., 2013). CD34 regulates angiogenesis in human pituitary adenomas and is overexpressed in aggressive pituitary tumors (Quah et al., 2021; Zhou et al., 2022). PTTG is an integrin heterodimeric receptor and plays a beneficial role in gathering the cell membrane with the extracellular matrix. PTTG has also been implicated in prolactinoma formation (Sánchez‐Ortiga et al., 2010; Sapochnik et al., 2016). FGFR4 is a member of the FGF family critical for pituitary organogenesis and progenitor cell proliferation during embryonic development and is widely expressed in invasive human pituitary adenomas (Abbass et al., 1997; Ezzat et al., 2002; Qian et al., 2004). Matrix metalloproteinases (MMPs) are zinc‐dependent proteinases that degrade the extracellular matrix, facilitating tumor invasion and metastasis. MMP‐9 overexpression correlates with higher invasive grades in human pituitary adenomas (Liu et al., 2016; Valea et al., 2022). Using immunohistochemistry, western blotting, and real‐time PCR, we found that PAE male rat pituitaries exhibited increased protein and mRNA expression of all these oncogenes. In addition, PAE tumors showed elevated N‐cadherin and reduced E‐cadherin expression, mirroring findings in human lactotropic tumors (Øystese et al., 2022; Trouillas et al., 2019; Valea et al., 2022). It is important to note that some cellular molecules were not significantly different between PF and AF rats but were different between AD and AF rats. PF dams received a comparable amount of liquid diet as did AF dams throughout gestation. It is possible that the stress related to prenatal diet manipulation is partially responsible for some of the observed effects in the current study. Despite this, this mode of alcohol consumption provides a valid model for various alcohol‐induced metabolic, endocrine, and central nervous system abnormalities (Alharshawi & Aloman, 2021; Lieber & DeCarli, 1989).

In conclusion, our data indicate that pituitary cells from PAE male rats exhibit increased expression of stemness markers, enhanced proliferation, colony formation, and migration, and successfully form tumors when transplanted into immunodeficient mice. These findings suggest that estrogen treatment induces aggressive prolactinomas in the pituitary gland of male rats following prenatal alcohol exposure.

## FUNDING INFORMATION

This work was supported by a National Institute of Health grant R01AA011591.

## CONFLICT OF INTEREST STATEMENT

No conflict of interest.

## Data Availability

The data that support the findings of this study are available from the corresponding author upon reasonable request.
